# Circulating miR-22 Early Predicts TACE Non-Response and Targets WEE1 in Hepatocellular Carcinoma

**DOI:** 10.3390/cells15080722

**Published:** 2026-04-19

**Authors:** Laura Gramantieri, Clara Vianello, Ilaria Leoni, Giuseppe Galvani, Elisa Monti, Marco Bella, Giorgia Marisi, Irene Salamon, Manuela Ferracin, Gloria Ravegnini, Catia Giovannini, Claudio Stefanelli, Maria Laura Lazzari, Fabio Piscaglia, Camelia A. Coada, Cristian Bassi, Massimo Negrini, Andrea Casadei-Gardini, Giuseppe Francesco Foschi, Davide Trerè, Francesca Fornari

**Affiliations:** 1 Division of Internal Medicine, Hepatobiliary and Immunoallergic Diseases, IRCCS Azienda Ospedaliero-Universitaria di Bologna, 40138 Bologna, Italy; catia.giovannini4@unibo.it (C.G.); fabio.piscaglia@unibo.it (F.P.); 2 Department for Life Quality Studies, University of Bologna, 47921 Rimini, Italy; clara.vianello2@unibo.it (C.V.); ilaria.leoni5@unibo.it (I.L.); giuseppe.galvani2@unibo.it (G.G.); elisa.monti10@unibo.it (E.M.); marco.bella3@unibo.it (M.B.); claudio.stefanelli@unibo.it (C.S.); 3 Biosciences Laboratory, IRCCS Istituto Romagnolo per lo Studio dei Tumori (IRST) “Dino Amadori”, 47014 Meldola, Italy; giorgia.marisi@irst.emr.it; 4 IRCCS Azienda Ospedaliero-Universitaria di Bologna, 40138 Bologna, Italy; irene.salamon@aosp.bo.it (I.S.); manuela.ferracin@unibo.it (M.F.); gloria.ravegnini2@unibo.it (G.R.); davide.trere@unibo.it (D.T.); 5 Department of Medical and Surgical Sciences, University of Bologna, 40138 Bologna, Italy; 6 Department of Farmacy and Biotechnology, University of Bologna, 40126 Bologna, Italy; 7 Department of Internal Medicine, Degli Infermi Hospital, AUSL Romagna, 48018 Faenza, Italy; marialaura.lazzari@auslromagna.it (M.L.L.); francesco.foschi@auslromagna.it (G.F.F.); 8 Department of Morpho-Functional Sciences, University of Medicine and Pharmacy “Iuliu Hațieganu”, 400006 Cluj-Napoca, Romania; coada_camelia_alexandra@elearn.umfcluj.ro; 9 Department of Translational Medicine, Laboratorio per le Tecnologie delle Terapie Avanzate (LTTA) Centre, University of Ferrara, 44100 Ferrara, Italy; cristian.bassi@unife.it (C.B.); massimo.negrini@unife.it (M.N.); 10 Department of Oncology, IRCCS San Raffaele Scientific Institute Hospital, Vita-Salute San Raffaele University, 20132 Milan, Italy; casadeigardini.andrea@hsr.it

**Keywords:** HCC, miR-22, TACE, WEE1

## Abstract

**Highlights:**

**What are the main findings?**

- Higher serum miR-22 levels are detected in non-responder patients two days after locoregional treatment, representing an early predictor of TACE resistance in HCC.
- The G2/M checkpoint kinase WEE1 is a miR-22 target and is involved in TACE resistance by regulating the DNA damage response.

**What are the implications of the main findings?**

- Early increases in circulating miR-22 after TACE identify HCC patients unlikely to respond to locoregional therapy.
- MiR-22/WEE1 axis is a potential therapeutic target to improve TACE effectiveness in intermediate-stage HCCs.

**Abstract:**

Transarterial chemoembolization (TACE) is the standard treatment for patients with intermediate-stage hepatocellular carcinoma (HCC), yet nearly half of treated patients fail to achieve durable benefit, and reliable biomarkers enabling early therapeutic stratification are still lacking. Treatment response is typically assessed by imaging one month after TACE and at three-month intervals, potentially delaying timely access to alternative therapies in non-responding patients. Circulating microRNAs (miRNAs) represent promising biomarkers due to their stability in body fluids and ease of detection. Here, we evaluated circulating miR-22 as an early predictor of TACE non-responder status and as a mechanistically relevant therapeutic target. Circulating miR-22 levels were measured by microarray and quantitative RT–PCR in three independent cohorts of early-to-intermediate-stage HCC patients undergoing TACE. Circulating miR-22 increased significantly in non-responders as early as 48 h after treatment, and fold changes consistently predicted treatment failure across two independent validation cohorts. Mechanistically, we identified the G2/M checkpoint kinase WEE1 as a direct functional target of miR-22. Modulation of the miR-22/WEE1 axis affected cell-cycle progression, proliferation, apoptosis, and DNA damage response in HCC cell lines and xenograft models. Under hypoxia-mimicking conditions combined with doxorubicin exposure, pharmacological inhibition of WEE1 induced mitotic catastrophe in highly proliferative miR-22-silenced cells. Collectively, these findings identify early post-TACE elevation of circulating miR-22 as a biomarker of non-response and highlight the miR-22/WEE1 axis as a potential target for precision treatment strategies in HCC.

## 1. Introduction

Primary liver cancer represents the third cause of cancer-related deaths worldwide, with hepatocellular carcinoma (HCC) accounting for approximately 90% of cases [1].

While early stages can benefit from curative options, intermediate stages, as well as early HCCs not amenable to curative approaches, are treated by transarterial chemoembolization (TACE), according to clinical practice [2]. Tumor cell killing by TACE is obtained through the delivery of chemotherapeutic drugs emulsified with lipiodol or loaded on embolizing beads directly in the HCC feeding artery, causing selective interruption of blood supply. Response to TACE is achieved in a variable percentage of cases in the different studies, reaching 52.5–57.1%, with an overall survival (OS) of 70.3–84.8% at one year, and 51.8–56.2% at two years [3,4]. Since objective response is an independent prognostic marker of survival in patients treated by TACE [5,6], the early prediction of response is of utmost importance to tailor subsequent follow-up and to refer patients to other approaches. Remarkably, in clinical practice, TACE is also used in early-stage HCC as a bridge to liver transplantation, or in down-staging protocols, also in keeping with the stage migration strategy [7]. In all these settings, non-invasive upfront and early biomarkers predictive of response might be of great help to personalize the post-treatment follow-up and to design individualized strategies, aimed at early switching to other options, when feasible, thus optimizing efficacy of treatments and avoiding losing time and liver function deterioration.

Circulating microRNAs (miRNAs) are promising diagnostic and predictive biomarkers in HCC [8]. An outstanding study by Wei et al. demonstrated the influence of the miR-125b/HIF1α axis in the selection of TACE-resistant CD24-positive cancer stem cells showing erythropoietin (EPO) overexpression [9]. Noticeably, high EPO levels before TACE were related to poor response, suggesting EPO as a surrogate marker for miR-125b/HIF1α axis deregulation, guiding clinicians for indication to TACE treatment in specific HCC subgroups. Another study reported the prognostic role of miR-21 ratio (after/before drug-eluting bead (DEB)-TACE) and miR-122 levels in predicting progression-free survival (PFS), identifying a positive correlation between circulating miR-21 and HIF1α levels in DEB-TACE-treated HCC patients [10]. Furthermore, high pre-treatment serum miR-221 levels independently predicted TACE refractoriness and overall survival in HBV-related HCC patients from a South-East Asian cohort [11]. Notably, Fako et al. identified a 15-gene signature associated with TACE resistance in different HCC cohorts and observed increased HIF1α and VEGFA levels in non-responder patients, with most of the TACE-related signature genes as direct targets of HIF1α [12].

Due to the intrinsic nature of miRNAs, they can be investigated not only as biomarkers, but also as possible therapeutic targets, being able to modulate the expression of hundreds of transcripts at the same time. For instance, Hu and coworkers demonstrated that miR-22 gene therapy outperforms lenvatinib in terms of OS in RAS/AKT-induced HCC mice, without noticeable toxicity [13]. Additionally, miR-22 influences the tumor immune microenvironment by expanding cytotoxic T cells and reducing regulatory T cells. We previously reported that circulating miR-22 is a promising biomarker of sorafenib response in advanced cases and is responsible for modulating HIF1α and metabolic reprogramming of HCC cells through GLUT1 targeting, contributing to drug resistance [14]. In particular, we observed that HCCs with high miR-22 expression downregulated pathways associated with G2/M phase transition, a crucial aspect in chemotherapy-mediated cancer cell killing.

WEE1 is a serine/threonine kinase that regulates the G2/M transition by phosphorylating CDK1, preventing premature mitotic entry and maintaining genomic stability. WEE1 upregulation was reported to counteract replication stress in many cancers, making it an interesting target for therapy [15]. Indeed, by increasing DNA damage and inducing mitotic catastrophe, WEE1 inhibition enhances the effectiveness of chemotherapies and anticancer drugs (e.g., PARP inhibitors). Despite this, the mechanisms underlying WEE1 deregulation in HCC are lacking, as well as its possible contribution to TACE resistance.

Here, we investigated circulating miR-22 as a possible predictive biomarker of response to TACE in early-intermediate HCC and dissected molecular events downstream of miR-22 deregulation during locoregional treatments, ultimately leading to the identification of pathogenic mechanisms, including WEE1 targeting, and hence, possible therapeutic targets to increase treatment efficacy.

## 2. Materials and Methods

### 2.1. Patients and Study Design

Three patient cohorts with early-intermediate HCC treated by TACE, both conventional (cTACE) and DEB-TACE, were tested to identify possible circulating miRNAs predicting response. In all patients, HCC was assessed according to the Barcelona Clinic Liver Cancer (BCLC) staging system and treatment choice was undertaken in accordance with the clinical practice and EASL guidelines [16]. The response was evaluated by contrast-enhanced computer tomography (CT) or magnetic resonance imaging (MRI), according to the mRECIST [17], at 1 month after TACE and, thereafter, every 3 months. Serum was processed 1–2 h after blood was drawn and stored at −80 °C for circulating miRNA analysis, as previously described [18]. Clinical data were obtained at the time of hospital admission and during follow-up, registered in medical records and are summarized in Table 1.

A first exploratory cohort (Bologna 1 cohort), referred to as the discovery cohort, was used to perform a microarray analysis on serum samples to identify circulating miRNAs differentiating responders and non-responders to TACE. Patients (*n* = 15) were enrolled at the Division of Internal Medicine, Hepatobiliary and Immunoallergic Diseases of the IRCCS Azienda Ospedaliero-Universitaria di S. Orsola of Bologna, according to clinical practice. Patients were selected based on the absence of previous treatments, ongoing medications for comorbidities, and the presence of a single early-stage HCC nodule, according to the BCLC classification. All patients were fully informed about the procedures of the research and provided written informed consent, under protocol approved by the local research ethics committee (138/2015/O/Tess and 528/2021/Sper/AOUBo).

A second, consecutive cohort (Bologna 2 cohort), referred to as validation cohort 1, was then analyzed to validate the findings from the microarray analysis. This cohort was composed of 52 consecutive patients affected by early and intermediate-stage HCC not amenable to curative treatment enrolled at the Division of Internal Medicine, Hepatobiliary and Immunoallergic Diseases of the IRCCS Azienda Ospedaliero-Universitaria di S. Orsola of Bologna, according to clinical practice. This cohort was analyzed to validate the potential of serum miR-22 levels, measured in blood samples collected at baseline and 48 h after TACE, and their fold-change (FC) as predictors of treatment response. Patients were enrolled after being accurately informed about the modalities and procedures of the research and signing the written informed consent, approved by the local research ethics committee (138/2015/O/Tess and 528/2021/Sper/AOUBo). Among the 52 patients, at the one- and three-month imaging assessment, 29 patients (55.7%) showed a radiological response based on contrast-enhanced CT or MRI. At the three-month assessment, but not at the one-month CT, two patients displayed the appearance of one more sub-centimetric nodule far from the site of treatment (in a different liver lobe). They were considered responders since the treated nodule did not show any active disease, also at the six-month imaging. Indeed, six-month follow-up imaging in these two patients showed that, in one case, MRI classified the new nodule as a macro-regenerative nodule rather than HCC, whereas in the second case, the nodule remained unchanged on CT, and continued follow-up was recommended. The remaining 23 patients showed different degrees of disease persistence at the site of treatment and were considered non-responders. At the subsequent 6-month assessment, active disease was observed in 9 of the 29 previous responders, while the remaining 19 showed no evidence of disease activity. One patient was lost to follow-up at 6 months. A total of 114 serum samples were analyzed: 52 were collected immediately before the procedure, 52 at 48 h post-procedure, and the remaining 10 were evaluated in biological replicates in two patients and at additional timepoints for other two patients (6 h before treatment start or 72 h after TACE), to assess the reproducibility of the analytical procedures and the stability of serum miR-22 levels.

A third, validation cohort (Faenza&IRST cohort), referred to as validation cohort 2, was subsequently investigated to validate the results from the discovery cohort and the validation cohort 1. This cohort was composed of 84 consecutively enrolled unselected HCC patients, assessed and treated with the same criteria as the Bologna cohorts. Patients were enrolled after receiving full information about the study procedures and providing written informed consent, under a protocol approved by the local research ethics committee (IRSTB041). Among the 84 patients, 46 (54.8%) were responders at one and three-month imaging control, while 38 patients were non-responders. At the 6-month assessment, disease reactivation was diagnosed in 7 of the 46 responders at three months, while 2 patients were lost at follow-up. The post-treatment blood sample was not available in 12 of 84 cases, equally distributed among responders (6 patients) and non-responders (6 patients); 32 non-responders and 40 responder patients were tested. A total of 156 serum samples were analyzed: 84 samples were obtained immediately before the procedure, and the remaining 72 at 48 h after the procedure. The missing samples were excluded from the post-treatment analyses. To test the robustness of our data, serum miR-22 levels were also assayed by droplet digital PCR (ddPCR) in post-treatment samples. In addition, 25 out of 84 patients were subjected to a second TACE cycle (25 and 23 samples were collected before and after the second TACE, respectively), whereas 9 out of 25 patients were subjected to a third TACE cycle (samples were collected before and after the third TACE for all 9 patients).

The bioinformatic analysis of data from The Cancer Genome Atlas Liver Hepatocellular Carcinoma (TCGA-LIHC) cohort is described in the Supplementary Materials, and the analysis is reported in Supplementary Datas S1 and S2.

### 2.2. Microarray Analysis

Total RNA was isolated from 400 μL of serum by using Trizol reagent (Thermo Fisher Scientific, Waltham, MA, USA), according to the manufacturer’s protocol. Circulating RNAs from 15 HCC serum samples (9 non-responders and 6 responders) were hybridized on Agilent whole-genome miRNAs microarray (G4470B, Agilent Technologies, Santa Clara, CA, USA) as previously described [18]. The microarray data generated and analyzed during the current study are available in the NCBI Gene Expression Omnibus (GEO) data repository with the accession number GSE327216.

### 2.3. Real-Time PCR and Droplet Digital PCR

For serum and cell supernatant samples, total RNA was extracted from 200 μL of serum/supernatant using the miRNeasy Mini Kit (QIAGEN, Venlo, The Netherlands) as previously described [18]. A synthetic miRNA (12.5 fmol), whose sequence does not match any known human small RNA sequence (cel-miR-39, Ambion, Waltham, MA, USA), was added to serum/supernatant samples after Qiazol addition. Its quantification by real-time PCR was used for data normalization and circulating miR-22 levels calculation by using the 2^−∆∆Ct^ method. Fold-change represents the ratio between post- and pre-treatment serum miR-22 levels. Exosomes were obtained by ultracentrifugation as previously described [18]. For cell pellets, total RNA was extracted with Trizol according to the manufacturer’s instructions. TaqMan MicroRNA Assays (Thermo Fisher Scientific) were used to evaluate miR-22-3p expression (ID 000398). RNU6B (ID 001093) was used as a housekeeping gene for intracellular miRNAs, while cel-miR-39 (ID 000200) was used as a spike-in for circulating miRNAs [18]. PowerUP SYBR-green (Thermo Fisher Scientific) was used for gene expression analysis by real-time PCR, using β-Actin as a housekeeping gene (Supplementary Table S1). Real-time PCR was run in triplicate.

Droplet digital PCR (ddPCR) with TaqMan assays was performed by using the QX200 Droplet Digital system (Bio-Rad, Hercules, CA, USA) as described by Miotto et al. [19].

### 2.4. Western Blot

Antibodies for Western blot (WB) analysis are reported in Supplementary Table S2. Digital images were quantified with ChemiDoc XRS+ (Image Lab Software, version 6.1, Bio-Rad, Hercules, CA, USA). Two independent experiments were performed. β-Actin and GAPDH were used as housekeeping genes.

### 2.5. HCC Cell Lines

HCC-derived cell lines were cultured as previously described [20], and the establishment of miR-22-overexpressing or silenced HCC cells was detailed in Leoni et al. [14]. WEE1 inhibition was obtained by treating cells with 400 nM adavosertib (AZD1775; Selleck Chemicals, Houston, TX, USA). Cells were treated with 0.25–0.5 µM doxorubicin-HCl (DOX-NP; Merck, Darmstadt, Germany) or 100 µM CoCl_2_ (Selleck Chemicals) or incubated in a hypoxic chamber with 1% O_2_ (InvivO_2_; Ruskinn Technology, Bridgend, UK). For the evaluation of extracellular miR-22 levels, treatments were performed in Opti-MEM I reduced serum medium (Thermo Fisher Scientific).

Incucyte Live-Cell Analysis System (Sartorius, Gottingen, Germany) was used for cell growth monitoring as previously detailed [21]. Apoptotic cell death and cell viability were evaluated by Caspase-Glo 3/7 and Cell-titer-Glo assays (Promega, Madison, WI, USA) according to manufacturer protocols. Each experiment was performed in quadruplicate. For the colorimetric viability assay, cells were fixed in paraformaldehyde (2% in PBS) for 10 min at room temperature (RT), stained with crystal violet (0.5% in 25% methanol) for 20 min at RT. Cells were resuspended in 10% acetic acid and measured at 595 nm. This experiment was performed in triplicate.

Cell cycle and BrdU (BD Bioscience, Franklin Lakes, NJ, USA) analyses were performed in duplicate by using Cytoflex S (Beckman Coulter, Brea, CA, USA) according to the manufacturer’s instructions. For cell-cycle analysis, cells were fixed in 70% ethanol at −20 °C for 16 h, washed once with PBS, resuspended in 500 μL PBS containing 10 μg/mL propidium iodide and 50 μg/mL RNase A and incubated for 30 min at room temperature. Stained cells were resuspended in 500 μL of PBS.

### 2.6. Luciferase Reporter Assay

WEE1 (GeneID: 7465) 3′-UTR vector (SC212987) was from Origene (Rockville, MD, USA). Mutagenesis of the miR-22 site was performed using the Phusion Site-Directed Mutagenesis Kit (Thermo Fisher Scientific) and verified by Sanger sequencing. Primers are reported in Supplementary Table S1. Reporter assay was performed by using Dual-Glo luciferase assay (Promega). Analysis was performed in two independent experiments in triplicate.

### 2.7. HCC Animal Models

The xenograft mouse (NOD/SCID females) model was obtained by subcutaneous injection of 5.0 × 10^6^ Huh-7 cells (pMXs-shRNA and pMXs-22) and 6.0 × 10^6^ HepG2 cells (MZIP-shRNA and MZIP-22) into both animal flanks. Five animals per group were used. To mimic TACE treatment, HepG2-derived xenograft mice (*n* = 5, injected into both flanks) were treated biweekly with dimethyloxalylglycine (DMOG), a HIF1α hydroxylase inhibitor, at a dose of 240 mg/kg by intraperitoneal injection for 3 weeks [22], starting when tumor volume reached 25–30 mm^3^. The day after the first DMOG injection, a single doxorubicin administration was performed by intravenous injection (tail vein), at a dose of 1 mg/kg. Tumor size was measured biweekly by caliper, and tumor volume was calculated by the following formula: *V* = (*D* ∗ *d*^2^)/2, with *D* = major diameter and *d* = minor diameter. A preliminary pilot study was conducted on three animals to test the efficacy of DMOG on HIF1α pathway activation. The control group (*n* = 1) was treated with PBS while the experimental group (*n* = 2) was treated biweekly with DMOG, at a dose of 240 mg/kg by intraperitoneal injection for 3 weeks. This protocol was approved by the Italian Minister of Health (N. 38/2022-PR).

### 2.8. Immunofluorescence

MiR-22-silenced HepG2 cells (MZIP-22) and control cells (MZIP-shRNA) were seeded at 2 × 10^5^ cells/well in 6-well plates with cover slips. The immunofluorescence (IF) protocol was performed after treatment with 100 µM CoCl_2_ and 0.5 µM doxorubicin for 24 h. For rescue experiments, miR-22-silenced HepG2 cells were treated with adavosertib (400 nM) or vehicle control (DMSO) for 24 h, followed by treatment with CoCl_2_ and doxorubicin for an additional 24 h.

Prior to fixation, cells were kept for 10 min in a pre-fixing solution consisting of 50% growth medium and 50% fixing solution. Cells were then fixed by adding ice-cold methanol:acetic acid (3:1, *v*/*v*) directly to the wells. After 10 min, the fixing solution was removed, and cells were permeabilized with PBS 1× containing 0.1% Triton X-100 (Merck, Rahway, NJ, USA) for 3 min. The wells were then washed with PBS 1× and incubated for 1 h with blocking solution (2% skimmed milk in PBS 1×), with gentle shaking. Primary antibody phospho-γ-H2AX (MA5-33062; Thermo Fisher Scientific) was diluted 1:2000 in blocking solution, added to coverslips and incubated for 2 h at RT without shaking. The secondary antibody CyTM3-conjugated Ab (Jackson ImmunoResearch Laboratories, Ely, UK) was diluted 1:200 in blocking solution and incubated for 1 h in the dark. For nuclear staining, DAPI (Thermo Fisher Scientific) was diluted 1:1000 in distilled water and incubated in the dark for 10 min. Between each incubation, coverslips were washed three times with PBS 1×. Finally, coverslips were mounted using Mount Quick Aqueous (Bio-Optica, Milan, Italy) and let dry under the hood for 20 min. Images were acquired with the IXploreSpinSR System (Evident, Tokyo, Japan) and analyzed with OlyVIA software, version 3.8 and Fiji ImageJ (NIH, Bethesda, MD, USA). Cells were considered positive when at least three γ-H2AX foci were present. At least eight images were analyzed, for a total of 300 cells per condition.

### 2.9. Statistical Analysis

Mann–Whitney was used to compare miR-22 circulating levels in responder and non-responder patients at the baseline and 48 h after TACE, along with miR-22 early FC from pre- to post-treatment. Spearman correlation was used to investigate any correlation between circulating miR-22 at the baseline, after TACE, or FC, and baseline AFP and HCC nodule size. Chi-square test was used to correlate the type of response and miR-22 early changes (FC) in responders and non-responders. Receiver operating characteristics (ROC) curves were used to evaluate the performance of miR-22 early changes (FC) as a possible predictive biomarker of response to TACE. Sensitivity, specificity, and area under the curve (AUC) were calculated for miR-22 baseline and after treatment levels, for miR-22 early FC and for AFP, used as a reference biomarker. The Kruskal–Wallis test was used to compare pre-miR-22 levels between different TACE cycles. Student’s *t*-test was used to evaluate differences between two groups in preclinical models. Statistical analyses were performed using SPSS Statistics version 20 (IBM, Tokyo, Japan) and GraphPad software version 8.0 (GraphPad Software, Boston, MA, USA). Statistical significance was defined as a two-sided *p*-value < 0.05. * *p* < 0.05, ** *p* < 0.01, *** *p* < 0.001, **** *p* < 0.0001.

## 3. Results

### 3.1. Identification of a miRNA Signature Associated with TACE Response in HCC Patients

**Figure 1:**
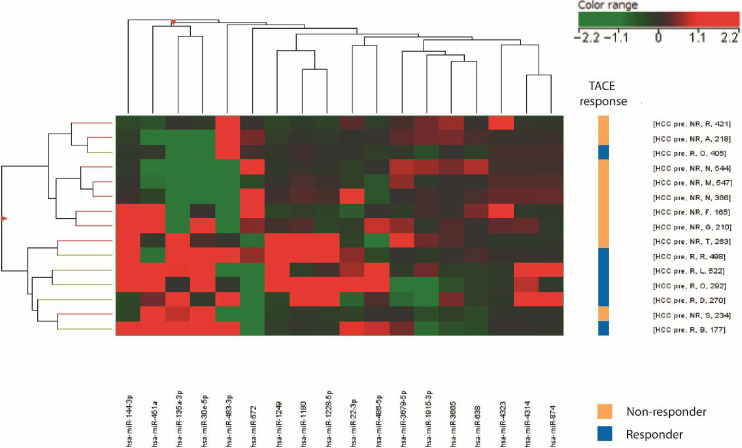
**Microarray analysis of HCC patients subjected to TACE.** Unsupervised hierarchical clustering of non-responder (NR, *n* = 9) and responder (R, *n* = 6) HCC patients (discovery cohort) based on the 18-miRNA signature. This analysis was performed in pre-treatment serum samples. Rows, biological samples; columns, miRNAs. For each miRNA, red means a higher expression value than its average expression across all samples, and green color means a lower expression value. Responder patients are represented by light blue squares and non-responders by orange squares.

An exploratory microarray analysis was performed in the discovery cohort of 15 patients with early-intermediate-stage HCC undergoing TACE. According to imaging assessments at one and three months after TACE, they were categorized as responders (*n* = 6) and non-responders (*n* = 9). Serum samples were collected before treatment. A supervised approach was used to identify miRNAs differentially expressed between the two groups, resulting in an 18-miRNA signature (Figure 1). Out of the significant miRNAs emerging from the microarray study, we focused on the tumor suppressor miR-22-3p (hereafter referred to as miR-22), which we previously demonstrated to be downregulated in HCC, associating with poor survival, metabolic reprogramming and response to sorafenib [14]. Interestingly, the microarray study detected a downregulation of pre-treatment miR-22 levels in most of the non-responder patients, suggesting miR-22 baseline downregulation as a possible predictor of non-response in patients subjected to TACE. We decided to focus on non-response because it may provide a more clinically actionable framework, enabling timely therapeutic redirection and personalized interventions. In light of this analysis, we subsequently investigated serum miR-22 levels as an upfront and early predictive biomarker of response to TACE in two independent validation cohorts.

### 3.2. Serum miR-22 Levels Predict TACE Response in HCC Patients

**Figure 2:**
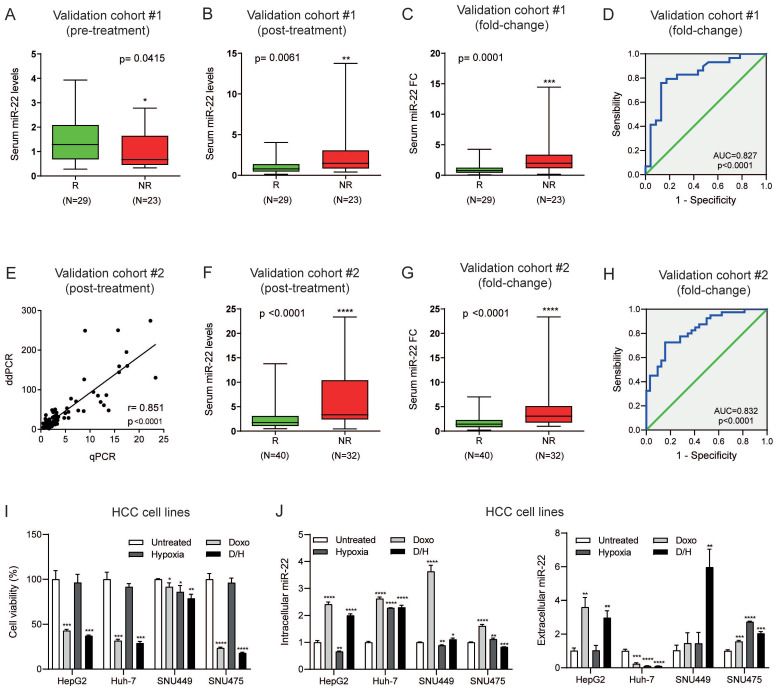
**Circulating miR-22 levels predict TACE response in HCC.** (**A**) Box plot graph of basal (pre-treatment) serum miR-22 levels in HCC patients (validation cohort 1) undergoing TACE. (**B**,**C**) Box plot graphs of post-treatment (48 h) and fold-change (FC) serum miR-22 levels in the same patients. Patients are divided into responders (R) and non-responders (NR) to TACE. Y-axes report 2^−∆∆Ct^ values corresponding to circulating miR-22 levels. Cel-miR-39 was used as a spike-in gene for miR-22 quantification by real-time PCR. Fold-change is the ratio between post- and pre-treatment miR-22 levels. (**D**) ROC curve showing the performance of serum miR-22 fold-change (FC) as a candidate biomarker of TACE response in validation cohort 1. (**E**) Correlation graph between qPCR and ddPCR measurements of post-treatment serum miR-22 levels in HCC patients (validation cohort 2) undergoing TACE. X-axis reports 2^−∆∆Ct^ values corresponding to miR-22 levels, while the Y-axis reports miR-22 copy number (copies/µL). Pearson’s correlation coefficient (*r*) was used. (**F**) Box plot graph of post-treatment (48 h) serum miR-22 levels in HCC patients (validation cohort 2) undergoing TACE. (**G**) Box plot graph of serum miR-22 fold-change (FC) between post and pre-treatment measurements in HCC patients (cohort 2) undergoing TACE. Patients are divided into responders (R) and non-responders (NR) to TACE. Y-axes report 2^−∆∆Ct^ values corresponding to circulating miR-22 levels. Cel-miR-39 was used as a spike-in gene for miR-22 quantification by real-time PCR. Fold-change is the ratio between post- and pre-treatment miR-22 levels. (**H**) ROC curve showing the performance of serum miR-22 fold-change (FC) as a candidate biomarker of TACE response in validation cohort 2. Mann–Whitney U-test was used to compare two independent groups of patients. Pearson correlation was used to measure the relationship between two variables. (**I**) Cell viability assay in HCC cell lines subjected to doxorubicin (doxo), hypoxia and doxorubicin plus hypoxia (D/H) treatments. The Y-axis reports percentage values corresponding to cell viability normalized on untreated cell lines. (**J**) Real-time PCR quantification of intracellular and extracellular miR-22 levels in HCC cell lines subjected to doxorubicin (doxo), hypoxia and doxorubicin plus hypoxia (D/H) treatments. Y-axes report 2^−∆∆Ct^ values corresponding to miR-22 levels normalized on untreated cell lines. Mean ± SD values are displayed. Cel-miR-39 was used as a spike-in gene for extracellular miRNA levels. U6RNA was used as a housekeeping gene for intracellular miR-22 levels. Student’s *t*-test was used to compare two variables in HCC cell lines. * = *p* < 0.05; ** = *p* < 0.01; *** = *p* < 0.001; **** = *p* < 0.0001.

Validation cohort 1 was initially used to test the reproducibility of serum miR-22 determinations, which was confirmed in repeated blood samples from two patients (different aliquots and different real-time PCR runs) and in blood samples additionally collected 6 h before or 72 h after treatment from two more patients (Supplementary Figure S1A). Lower baseline serum miR-22 levels differentiated non-responder from responder patients (median in non-responders versus responders: 0.67 versus 1.29; Mann–Whitney, *p* = 0.042), confirming the microarray analysis (Figure 2A).

We also investigated circulating miR-22 levels in blood samples collected after locoregional treatment, hypothesizing that miRNA release due to tumor necrosis or active extrusion mechanisms might be informative of treatment response. At 48 h, higher serum miR-22 levels (median in non-responders versus responders: 1.48 versus 0.79; Mann–Whitney, *p* = 0.0061) predicted non-response to TACE at the three-month follow-up (Figure 2B). Next, we tested treatment-associated early variations in serum miR-22 levels expressed as fold-change (FC). Non-responder patients displayed an increase in miR-22 FC levels compared with responders (median in non-responders versus responders: 1.98 versus 0.74; Mann–Whitney, *p* < 0.001). Because miR-22 quantification was performed soon after TACE, the two responder patients with new sub-centimetric nodules at the 3-month follow-up were included in this analysis. Interestingly, 20 out of 23 (87%) non-responder patients displayed a miR-22 increase soon after the procedure, while three patients showed a miR-22 FC decrease. On the other hand, among the 29 responder patients, 22 (76%) showed a reduction in miR-22 FC while seven experienced an increase (Chi-square test, with Yates correction 17.84, *p* < 0.001). These findings outline the informativeness of an early circulating miR-22 increase as a possible predictor of non-response to TACE.

The performance of circulating miR-22 FC as a candidate of treatment response in patients undergoing TACE was further assessed by ROC curve analysis which confirmed the interesting findings of its early variation, showing an AUC of 0.827 (95% CI: 0.709–0.945) and a best cut-off value of 1.052, with a sensitivity of 0.870 and a specificity of 0.759 (Figure 2D). This marker performed better than baseline AFP, which displayed an AUC of 0.680 (95% CI: 0.521–0.839). According to clinical practice, AFP was not assayed 48 h after TACE; the 48 h AFP FC could not be compared with the miR-22 FC. Despite the direct correlations between miR-22 FC and miR-22 baseline levels (Spearman correlation R = 0.593, *p* = 0.001) and between miR-22 FC and miR-22 post-TACE levels (Spearman correlation R = 0.697, *p* = 0.001), neither baseline nor post-treatment serum miR-22 levels displayed a better performance than miR-22 FC in validation cohort 1, showing an AUC of 0.357 (95% CI 0.203–0.510) and 0.712 (95% CI 0.570–0.854), respectively. No correlation was found between serum miR-22 levels (baseline, after TACE and FC) and HCC size or baseline AFP. The increase in circulating miR-22 observed after TACE may reflect active extrusion mechanisms. Moreover, consistently with its tumor-suppressive role, higher post-treatment levels may indicate reduced intracellular miR-22 in tumor cells and thus adaptive resistance processes, supporting its potential as a predictor of treatment outcome.

To confirm the findings from validation cohort 1, we analyzed an additional independent cohort from a different center (validation cohort 2) comprising 84 patients with early- or intermediate-stage HCC treated with TACE. First, to assay the robustness of our data, we quantified serum miR-22 levels in post-treatment samples by ddPCR, a methodology often used to quantify circulating miRNAs in body fluids [19], showing a good correlation between the two techniques (Figure 2E).

While baseline miR-22 serum levels were not confirmed as an upfront predictive biomarker in validation cohort 2, at the 48 h post-treatment evaluation, higher miR-22 circulating levels predicted the 3-month non-response to TACE (median in non-responders versus responders: 3.34 versus 1.77; Mann–Whitney, *p* < 0.0001), confirming the data observed in validation cohort 1 (Figure 2F). In the same line, non-responder patients displayed higher miR-22 FC levels (median in non-responders versus responders: 3.08 versus 1.14; Mann–Whitney, *p* < 0.0001) compared to responders (Figure 2G), with 27 out of 32 (84%) non-responder patients showing an increase in miR-22 levels soon after the procedure, and the remaining five patients showing decreased miR-22 levels. On the other hand, among the 40 responders, 29 (73%) showed a reduction in miR-22 FC levels while 11 experienced an increase (Chi-square test, with Yates correction 20.85, *p* < 0.001). In this cohort as well, miR-22 levels did not correlate with baseline serum AFP values, which were available for 68 cases. The predictive performance of miR-22 FC as a marker of nonresponse to TACE was further confirmed by ROC curve analysis, showing an AUC of 0.832 (95% CI 0.740–0.924) with a sensitivity of 0.725 and a specificity of 0.844 (Figure 2H). This marker performed better than baseline AFP, which displayed an AUC of 0.584 (95% CI: 0.434–0.735).

At the univariate analysis, both tumor size and AFP levels predicted non-response to TACE (Supplementary Figure S1B,C). Thus, we performed multivariate analysis with miR-22 FC, tumor size and AFP. Notably, at multivariate logistic regression analysis, miR-22 FC levels were independently associated with TACE non-response (OR = 2.445, 95% CI: 1.295–4.618, *p* = 0.006), whereas tumor size (OR = 1.037, 95% CI: 0.975–1.102, *p* = 0.248) and AFP (OR = 5.074, 95% CI: 0.924–27.857, *p* = 0.062) did not reach statistical significance. No correlation was found between serum miR-22 levels (baseline, after TACE and FC) and HCC size. Despite this, decreased baseline miR-22 levels were observed in HCC patients undergoing subsequent TACE cycles (Supplementary Figure S1D). In addition, higher miR-22 post-treatment and FC levels were detected in non-responder patients undergoing the second TACE cycle (*n* = 23) compared to responders (Supplementary Figure S1E,F), further strengthening the robustness of our data.

Taken together, our findings indicate that higher post-treatment miR-22 levels and miR-22 FC can predict early non-response to TACE at 3 months. Given the simplicity and reproducibility of these measurements, and their availability as early as 48 h post-procedure, miR-22 FC emerges as a promising non-invasive early predictor of non-response to TACE in patients with HCC, warranting further investigations.

### 3.3. MiR-22 Is Secreted by HCC Cells in Response to Doxorubicin Treatment Under Hypoxic Conditions

MicroRNAs can be informative as biomarkers, but they also deserve attention as therapeutic targets, as shown for miR-122 restoration in HCV-infected patients [23]. Thus, to gain insight into the molecular events underlying miR-22 variations triggered by TACE, we reproduced the mechanisms by which TACE induces cell death in HCC cell lines by coupling doxorubicin treatment in the presence of a hypoxic microenvironment. In line with the tumor suppressor role of miR-22 in HCC [14], an inverse correlation between basal intracellular miR-22 levels and cell viability in the presence of doxorubicin treatment and hypoxia was observed in HCC cell lines (Supplementary Figure S1G). Similarly, a trend towards an inverse correlation was observed between intracellular miR-22 levels and tumor volume in xenograft mice treated with the HIF1α activator, DMOG, and doxorubicin (Supplementary Figure S1H). The activation of the HIF1α pathway by DMOG was demonstrated in a pilot study showing increased expression of its transcriptional target genes (Supplementary Figure S1I). This experimental model (Supplementary Figure S1J), employed to mimic TACE in vivo, further confirmed our hypothesis that reduced intratumor miR-22 levels, possibly due to increased miRNA extrusion, are associated with TACE resistance. Because of the difficulty of performing TACE in the animal model, we used DMOG to induce HIF1α activation, which is often observed after this locoregional treatment.

Next, we focused on investigating how doxorubicin and hypoxia modulate intracellular and extracellular miR-22 levels in HCC cell lines with different genetic and molecular backgrounds and different treatment susceptibility. Since HCC cell lines displayed variable responses to treatment, we focused on four cell lines exhibiting the lowest (SNU449), highest (SNU475), and intermediate (HepG2 and Huh-7) sensitivity to the combined treatment (Figure 2I). An increase in extracellular miR-22 levels following the combined treatment was observed in three out of four tested cell lines (Figure 2J). Interestingly, doxorubicin treatment and hypoxic conditions exerted the strongest synergistic effect in terms of miR-22 extrusion in SNU449 cells, which, displaying the lowest cell death, were considered the most resistant to the combination of doxorubicin and hypoxia, mirroring findings observed in non-responder patients undergoing TACE. Moreover, the high viability of SNU449 cells treated with Doxorubicin and CoCl_2_ (approximately 90% of untreated cells) suggests that the observed secretion is more likely due to an active mechanism rather than passive release from cancer cells. This assumption is further strengthened by the presence of a positive correlation between extracellular and exosomal miR-22 levels in eight HCC cell lines (Supplementary Figure S1J).

Taken together, these findings support miR-22 release as a potential mechanism of resistance to doxorubicin under hypoxic conditions, consistent with what was observed in patients who do not respond to TACE. This evidence lets us move towards uncovering the molecular mechanisms downstream of miR-22 modulation that are associated with response to doxorubicin under hypoxic conditions, finally aiming to identify a possible strategy to increase TACE efficacy.

### 3.4. WEE1 Is a Direct Target of miR-22 in HCC

**Figure 3:**
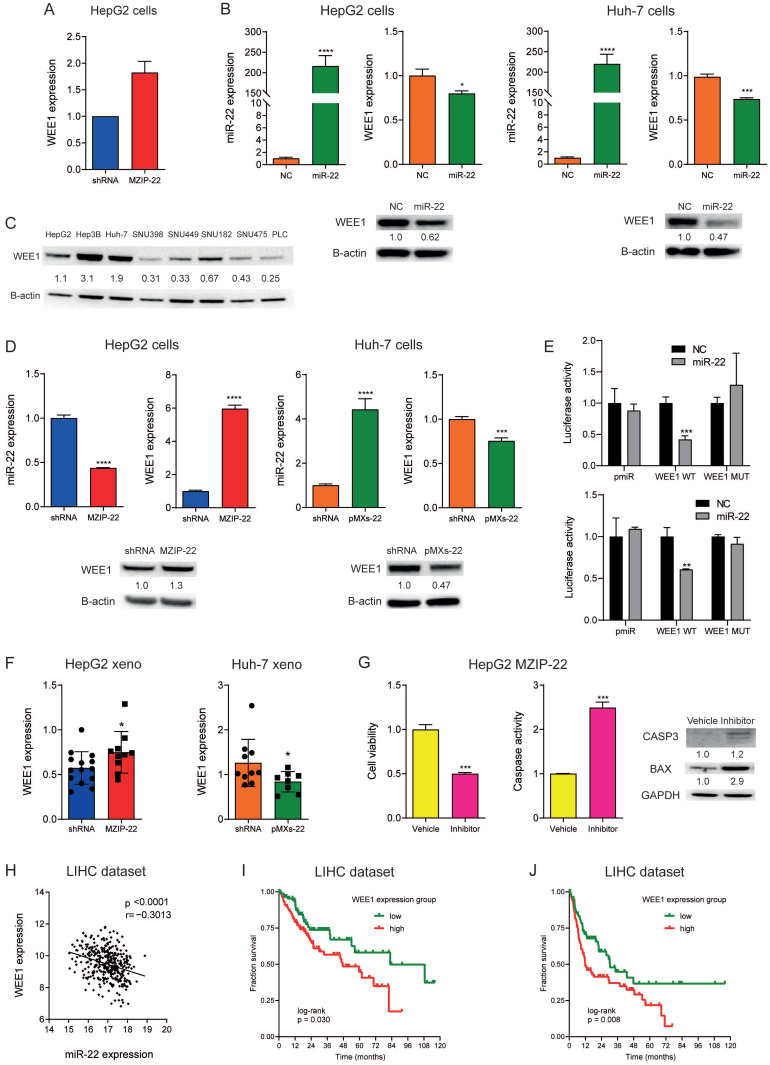
**WEE1 is a target of miR-22 and associates with poor prognosis in HCC.** (**A**) RNA sequencing analysis shows WEE1 downregulation in miR-22-silenced (MZIP-22) HepG2 cells compared with control cells (shRNA). This analysis was performed in duplicate. (**B**) Real-time PCR analysis of miR-22 and WEE1 expression and WB analysis of WEE1 expression in miR-22-overexpressing HCC cell lines. Y-axes report 2^−ΔΔCt^ values corresponding to miR-22 and WEE1 expression normalized to controls. Mean ± SD values are displayed. Beta-actin was used as a housekeeping gene for real-time PCR and WB analyses of WEE1 expression. (**C**) WB analysis of WEE1 expression in HCC cell lines. Beta-actin was used as a housekeeping gene. (**D**) Real-time PCR analysis of miR-22 and WEE1 expression and WB analysis of WEE1 expression in miR-22-silenced (MZIP-22) HepG2 cells and miR-22-overexpressing (pMXs-22) Huh-7 cells and related controls (shRNA). Y-axes report 2^−∆∆Ct^ values corresponding to miR-22 and WEE1 expression normalized to controls. Mean ± SD values are displayed. Beta-actin was used as a housekeeping gene for real-time PCR and WB analyses of WEE1 expression. (**E**) Dual-luciferase activity assay of wild-type (WT) and mutant (MUT) WEE1-3′UTR vectors co-transfected with miR-22 in HepG2 (top graph) and Huh-7 (bottom graph) cells. NC: Negative control precursor miRNA. Y-axes report the firefly/renilla ratio normalized to controls. Mean ± SD values are displayed. (**F**) Real-time PCR analysis of WEE1 expression in xenograft tumors (*n* = 10) derived from miR-22-silenced (MZIP-22; black circles) HepG2 cells and miR-22-overexpressing (pMXs-22; black squares) Huh-7 cells and matched controls (shRNA). Beta-actin was used as a housekeeping gene. Y-axes report 2^−∆∆Ct^ values corresponding to WEE1 expression normalized to controls. Mean ± SD values are displayed. (**G**) Cell viability and caspase activity assays and WB analysis of apoptotic genes in miR-22-silenced (MZIP-22) HepG2 cells treated with adavosertib (400 nM, 48 h). Vehicle: DMSO. Y-axis report chemiluminescent signals normalized to controls. Mean ± SD values are displayed. (**H**) Correlation graph between miR-22 and WEE1 mRNA levels in HCC patients from the “LIHC” cohort. Axes report values corresponding to miR-22 and WEE1 expression levels from RNA sequencing analysis. (**I**,**J**) Kaplan–Meier curves of overall survival and progression-free survival in HCC patients from the “LIHC” cohort with high (red line) or low (green line) WEE1 expression. Student’s *t*-test was used to compare two independent groups. Pearson correlation was used to measure the relationship between two variables. * = *p* < 0.05; ** = *p* < 0.01; *** = *p* < 0.001; **** = *p* < 0.0001.

Previous investigations in the “Liver Hepatocellular Carcinoma (LIHC)” dataset from The Cancer Genome Atlas (TCGA) comparing high versus low miR-22-expressing cases showed a negative enrichment for cell-cycle progression and hallmarks of G2/M checkpoint [14,24]. Considering these findings, we conducted a bioinformatics investigation identifying the G2 checkpoint kinase WEE1 among miR-22 targets (Figure S2A). We also performed an explorative RNA sequencing analysis in miR-22-silenced HepG2 cells compared to control cells, detecting an upregulation of WEE1 expression (Figure 3A). Moreover, a negative correlation between miR-22 and WEE1 was observed in HCC cell lines (Figure S2B), suggesting its possible regulation by miR-22.

To assess whether miR-22 regulates WEE1 expression, we transfected miR-22 precursor oligonucleotides into HepG2 and Huh-7 cells, which exhibit low basal miR-22 levels, consistent with miR-22 downregulation in HCC tissues [14]. A downregulation of WEE1 mRNA and protein levels was detected following miR-22 overexpression in both cell lines (Figure 3B). Subsequently, based on WEE1 protein levels in HCC cell lines (Figure 3C), we stably silenced miR-22 in HepG2 cells, which display low WEE1 expression, and overexpressed miR-22 in Huh-7 cells, characterized by high WEE1 expression. In line with transient transfection, miR-22-silenced HepG2 cells (MZIP-22) showed WEE1 upregulation, while miR-22-overexpressing Huh-7 cells (pMXs-22) showed WEE1 downregulation (Figure 3D). For the reporter assay, we cloned wild-type (WT) and mutant (MUT) WEE1 3′UTR downstream of the luciferase gene. A decrease in luciferase activity was detected in miR-22 and WT vector co-transfected HCC cells but not in MUT vector co-transfected cells, demonstrating the direct interaction between miR-22 and its complementary binding site in WEE1 mRNA (Figure 3E).

As previously reported, miR-22-silenced HepG2 cells produced larger tumors, whereas miR-22-overexpressing Huh-7 cells gave rise to smaller tumors [14]. Notably, in vivo findings in xenograft mice confirmed miR-22-mediated modulation of WEE1, with increased expression in miR-22-silenced HepG2-derived tumors and decreased expression in miR-22-overexpressing Huh-7-derived tumors (Figure 3F). To investigate whether miR-22 exerts its biological effect through WEE1 targeting, we performed rescue experiments with the WEE1 inhibitor adavosertib in miR-22-silenced HepG2 cells, which we previously reported to display increased cell viability and decreased apoptotic cell death compared to control cells [14]. As expected, we observed reversal of this phenotype following treatment with the WEE1 inhibitor, which resulted in decreased cell viability and increased caspase activity compared to control cells (Figure 3G). Finally, an inverse correlation between miR-22 and WEE1 was confirmed in the TCGA-LIHC dataset, highlighting the clinical relevance of miR-22-mediated regulation of WEE1 in human HCC (Figure 3H). The Kaplan–Meier analysis highlighted poorer OS and PFS for high versus low WEE1-expressing HCC patients from the LIHC cohort (Figure 3I,J), suggesting that the miR-22/WEE1 axis might play an active role in HCC aggressiveness.

### 3.5. MiR-22/WEE1 Axis Regulates Cell-Cycle Progression and Apoptosis in HCC Cells

**Figure 4:**
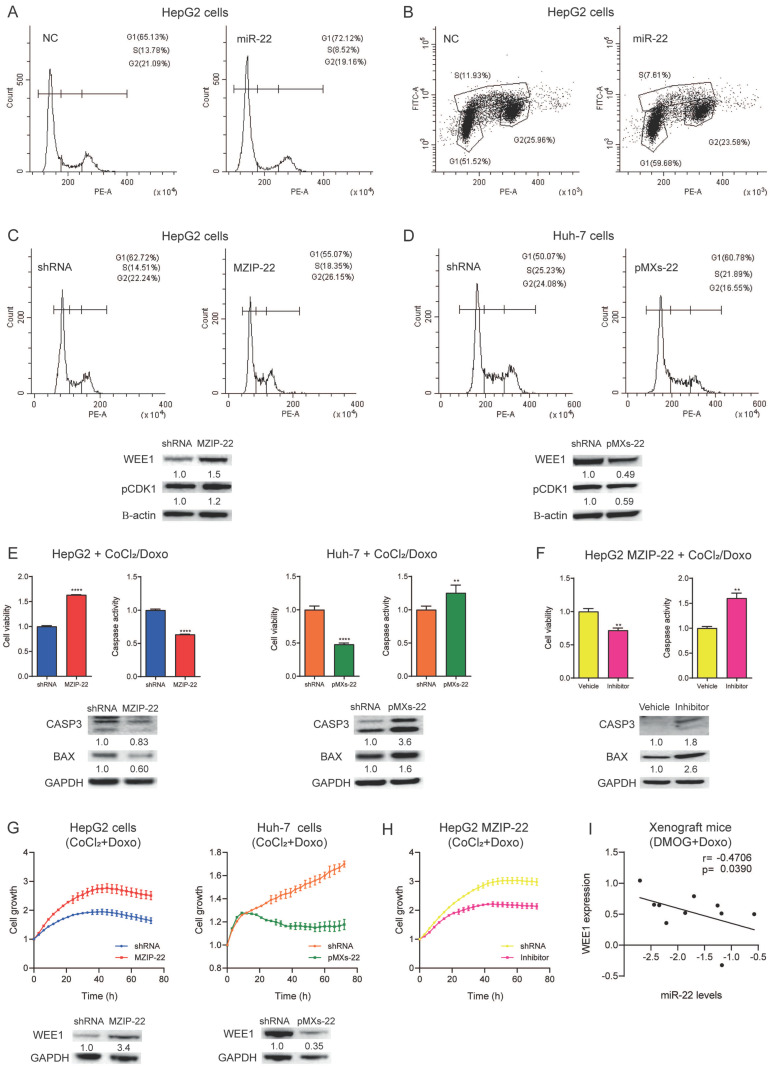
**The miR-22/WEE1 regulates cell-cycle progression, cell proliferation and apoptosis in HCC cells.** (**A**,**B**) Cell-cycle analysis and BrdU incorporation assay in miR-22-overexpressing and control (NC) HepG2 cells (48 h). (**C**,**D**) Cell-cycle analysis in miR-22-silenced (MZIP-22) HepG2 cells and miR-22-overexpressing (pMXs-22) Huh-7 cells and matched controls (shRNA). WB analysis of WEE1 and phospho-CDK1 expression in the same cells. Beta-actin was used as a housekeeping gene. (**E**) Cell viability, caspase activity assays and WB analysis of apoptotic genes in miR-22-silenced (MZIP-22) HepG2 cells and miR-22-overexpressing (pMXs-22) Huh-7 cells and matched controls (shRNA) treated with doxorubicin (0.5 µM) and CoCl_2_ (100 µM) for 48 h. Y-axis report chemiluminescent signals normalized to controls. Mean ± SD values are displayed. (**F**) Cell viability and caspase activity assays and WB analysis of apoptotic genes in miR-22-silenced (MZIP-22) HepG2 cells treated with WEE1 inhibitor (400 nM) or vehicle (DMSO) and subjected to doxorubicin (0.5 µM) and CoCl_2_ (100 µM) for 48 h. Y-axis report chemiluminescent signals normalized to controls. Mean ± SD values are displayed. (**G**) Growth curves of miR-22-silenced (MZIP-22) HepG2 cells and miR-22-overexpressing (pMXs-22) Huh-7 cells and matched controls (shRNA) treated with doxorubicin (0.5 µM) and CoCl_2_ (100 µM). WB analysis of WEE1 expression in the same cells. Beta-actin was used as a housekeeping gene. (**H**) Growth curves of miR-22-silenced (MZIP-22) HepG2 cells treated with WEE1 inhibitor (400 nM) or vehicle (DMSO) and subjected to doxorubicin (0.5 µM) and CoCl_2_ (100 µM). Growth curves were normalized to T0. Mean ± SD values are reported. (**I**) Correlation plot between intratumor miR-22 and WEE1 mRNA levels in doxorubicin plus DMOG-treated xenograft mice. Axes show log2-transformed 2^−ΔΔCt^ values corresponding to miR-22 and WEE1 levels. ** = *p* < 0.01; **** = *p* < 0.0001.

To demonstrate the active role of WEE1 in regulating the G2/M phase transition, we performed cell-cycle analysis and evaluated the phosphorylation of its downstream target CDK1 in our in vitro models. Consistent with WEE1 targeting, miR-22 overexpression determined a block in the G1 phase and a decrease in the S and G2 phases in HepG2 cells, as also confirmed by the BrdU analysis (Figure 4A,B). In stable cell lines, an increase in the G2 phase and CDK1 phosphorylation was detected in miR-22-silenced HepG2 cells, while a decrease in the G2 phase and CDK1 phosphorylation was observed in miR-22-overexpressing Huh-7 cells (Figure 4C,D).

We also evaluated the effect of the miR-22/WEE1 axis in regulating cell viability, apoptosis and cell growth under chemical hypoxia and doxorubicin treatment in in vitro models used to mimic TACE effects in HCC nodules. Increased cell viability and decreased apoptotic markers were observed in miR-22-silenced HepG2 cells following CoCl_2_/doxorubicin treatment, while the opposite was detected in miR-22-overexpressing Huh-7 cells (Figure 4E). To assess the contribution of WEE1, miR-22-silenced HepG2 cells were treated with CoCl_2_ and doxorubicin in the presence of adavosertib. This resulted in a reversal of the treatment-resistant phenotype, characterized by decreased cell viability and increased apoptotic cell death (Figure 4F). Real-time live-cell imaging revealed a higher cell growth rate in miR-22-silenced HepG2 cells treated with CoCl_2_/doxorubicin compared to control cells, whereas miR-22-overexpressing Huh-7 cells exhibited a lower growth rate compared to controls (Figure 4G). Adavosertib treatment in miR-22-silenced HepG2 cells reduced the cell growth rate, further reversing the treatment-resistant phenotype (Figure 4H). Of note, adavosertib had a stronger effect in miR-22-silenced HepG2 cells compared to control cells, increasing their doubling time by 3.0 times versus 1.8 times, respectively (Figure S2G). WEE1 regulation by miR-22 was also observed in the presence of CoCl_2_/doxorubicin treatment (Figure 4G). In line, an inverse correlation between intratumor miR-22 and WEE1 levels was detected in xenograft mice treated with DMOG and doxorubicin (Figure 4I), suggesting the relevance of miR-22-mediated WEE1 targeting in TACE-mimicking conditions in preclinical models.

On the one hand, these findings support the role of reduced intracellular miR-22 levels as a trigger of resistance against doxorubicin coupled with hypoxia by interfering with cell-cycle progression and inhibiting apoptotic cell death in a WEE1-dependent manner. On the other hand, they also suggest miR-22 replacement as an option to increase TACE effectiveness.

### 3.6. MiR-22/WEE1 Axis Regulates DNA Damage in Doxorubicin-Treated HCC Cells Under Hypoxia-like Conditions

**Figure 5:**
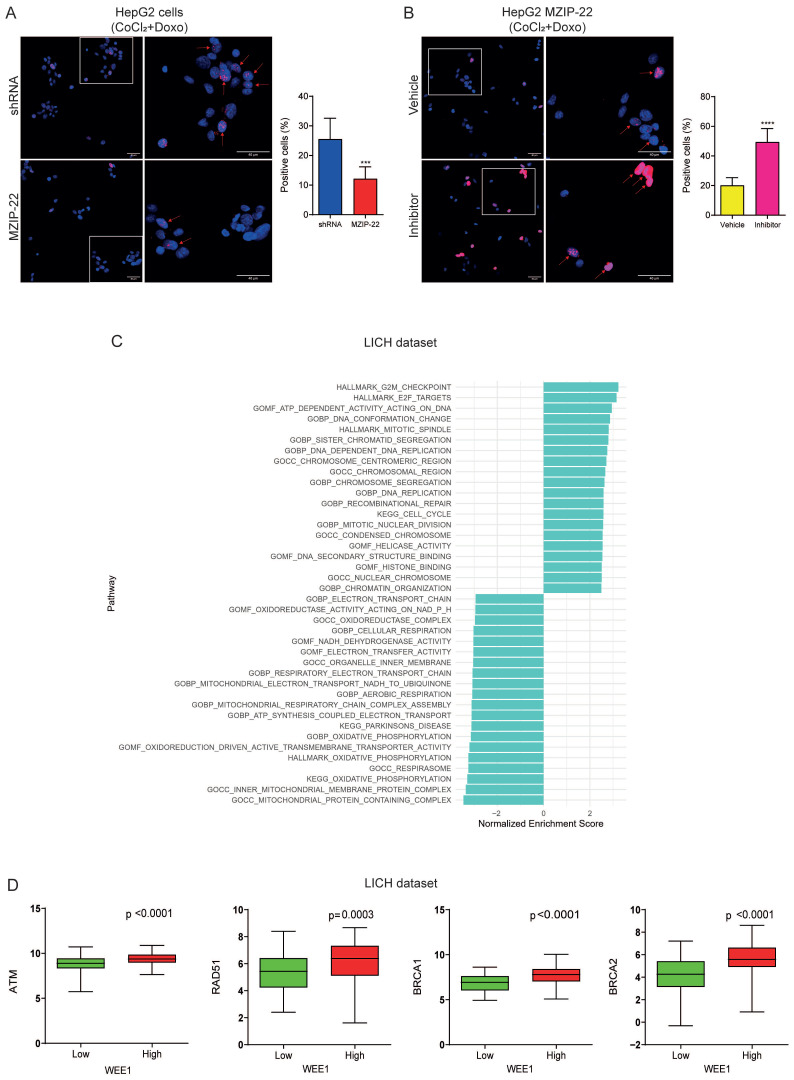
**The miR-22/WEE1 axis modulates the DNA damage response in HCC.** (**A**) Representative images of immunofluorescence staining of γ-H2AX nuclear foci (red signal) in miR-22-silenced (MZIP-22) HepG2 cells and control cells (shRNA) treated with doxorubicin (0.5 µM) and CoCl_2_ (100 µM) for 24 h. (**B**) Representative images of immunofluorescence staining of γ-H2AX nuclear foci (red signal) in miR-22-silenced (MZIP-22) HepG2 cells treated with the WEE1 inhibitor adavosertib (400 nM) or control vehicle (DMSO) for 24 h and then treated with doxorubicin (0.5 µM) and CoCl_2_ (100 µM) for an additional 24 h. Blue signal: DAPI nuclear staining. Red arrows indicate γ-H2AX foci-positive cells. The bar chart represents the percentage values of positive cells on total nuclei for each image. Three hundred cells were considered for each condition. Mean ± SD values are reported. Images (left) were acquired at 40× magnification (scale bar: 40 µm). A magnified view of the boxed area is shown in the right image. (**C**) Normalized enrichment score (NES) analysis of hallmark pathways in HCC patients with high versus low WEE1 expression (LIHC cohort). Gene set enrichment analysis (GSEA) using hallmark gene sets from the Molecular Signatures Database (MSigDB). Right bars indicate the pathways enriched in high WEE1-expressing patients, and left bars represent those enriched in low WEE1-expressing patients. The top-most statistically significant signatures were plotted. (**D**) Box plot graphs of DNA repair genes in high versus low WEE1-expressing HCC patients from the LIHC cohort. The y-axis reports gene expression. *** = *p* < 0.001; **** = *p* < 0.0001.

Because the G2 checkpoint kinase WEE1 plays a central role in chemotherapy-induced mitotic catastrophe by regulating the accumulation of mitotic stress, we assessed DNA damage by analyzing nuclear γ-H2AX foci in our in vitro models. Fewer foci were detected in miR-22-silenced HepG2 cells treated with CoCl_2_/doxorubicin compared with control cells (Figure 5A). Conversely, adavosertib treatment reversed the phenotype of miR-22-silenced HepG2 cells, resulting in a higher number of γ-H2AX foci (Figure 5B). In line, enriched genes and pathways involved in the G2/M phase transition, cell-cycle regulation, and DNA repair were identified in high versus low WEE1-expressing HCC patients from the LIHC cohort (Figure 5C and S2H), confirming preclinical findings. Notably, DNA repair genes (ATM, RAD51, BRCA1/2) were upregulated in high versus low WEE1-expressing patients (Figure 5D), likely contributing to TACE resistance in low miR-22-expressing HCCs.

Taken together, these findings demonstrate the involvement of the miR-22/WEE1 axis as a mechanism undermining doxorubicin-mediated DNA damage under hypoxic conditions.

## 4. Discussion

TACE represents the standard of care for early-intermediate HCC, as well as an option for tumor downstaging as a bridge to liver transplantation (OLT). Therefore, upfront and early post-treatment biomarkers predictive of treatment response could help tailor personalized strategies, enabling a timely transition to alternative approaches while avoiding delays and preserving liver function. In addition, biomarkers with a defined biological role may allow the identification of patient-specific vulnerabilities to be targeted by tailored treatment combinations. At present, no biomarkers help predict response to TACE.

Here, we report an early increase in circulating miR-22 levels in HCC patients who did not respond to TACE, occurring shortly after the procedure. This finding was confirmed in two independent cohorts of patients with early-intermediate HCC treated by TACE at two different centers. Notably, the study by Rinaldi et al. described not only the predictive value of cell-free miR-22-3p in serum samples from diffuse large B-cell lymphoma (DLBCL) patients treated with R-CHOP chemotherapy, but also its prognostic potential [25]. Higher serum miR-22 levels are associated with worse 2-year PFS, discriminating refractory from R-CHOP responder patients. Among non-invasive biomarkers, the study by Campani et al. reported a significant rise in cell-free DNA (cfDNA) 24 h after locoregional treatments, such as TACE and percutaneous ablation [26]. An increase in mutation detection was observed in post-treatment samples, showing a certain correspondence with the tumor tissue, even though most tumor mutations were still undetectable in the plasma, even at 24 h post-treatment. In the setting of predictive biomarkers and their dynamic changes, here we show that circulating miRNAs, such as miR-22, may represent an appealing option due to their informativeness and easy analytical assay.

On the one hand, our observations suggest that the early increase in serum miR-22 levels could be further explored as a promising non-invasive predictor of TACE response. On the other hand, our in vitro data suggest that miR-22 may be preferentially secreted by HCC cells with resistance to doxorubicin under hypoxic conditions (e.g., SNU449 cells), and that its overexpression in the tumor could be associated with increased treatment efficacy. In vivo, xenograft mice treated with the HIF1α activator DMOG and doxorubicin, used to mimic TACE in our model, exhibited a trend towards a negative correlation between intracellular miR-22 levels and tumor size. These findings support the hypothesis that miR-22 active secretion might reduce its intracellular levels, thus triggering WEE1 expression that desensitizes tumor cells to doxorubicin, undermining the TACE effect. Indeed, by blocking the G2/M phase transition, WEE1 allows DNA damage repair mechanisms to resolve DNA breaks, thereby protecting cells from apoptosis, as supported by increased expression of DNA repair genes in HCC patients with high WEE1 expression. The active secretion of miR-22 following hypoxia-inducing treatment, such as TACE, agrees with previous data showing an inverse correlation between intra- and extracellular miR-22 levels in the DEN-HCC rat model treated with the antiangiogenic agent sorafenib [14]. Preclinical findings reported in this study suggest that replacing miR-22 in the setting of TACE could be a rational strategy to increase treatment efficacy. In addition, the increase in miR-22 levels immediately after TACE in non-responder patients could represent not only an early biomarker of treatment resistance, but also a possible indication of replacing miR-22 in subsequent TACE retreatments, whether clinically indicated in selected patients.

Of note, Hu and colleagues [13] reported the safety and antitumor efficacy of systemic adeno-associated virus-mediated delivery of miR-22 in HCC mice, resulting in decreased tumor burden and inactivation of HIF1α, a well-known target of this miRNA [27], undoubtedly involved in TACE response [9]. The same authors also described the antitumor effect of miR-22 delivery in metabolic dysfunction-associated steatohepatitis (MASH)-HCC mouse models, in both preventive and curative settings, through the regulation of multiple metabolic and oncogenic pathways [24]. Given the multitargeting nature of miRNAs, we demonstrate for the first time that the pseudo-oncogene WEE1 is a direct target of miR-22 in HCC, uncovering a previously unrecognized role for this miRNA as a central regulator of pathways governing chemotherapy response under hypoxic conditions. In agreement with this, gene set enrichment analysis performed by Fako et al. showed that the G2/M checkpoint is the second most enriched pathway in responder versus non-responder HCC patients undergoing TACE, along with hypoxia- and metabolism-related pathways [12]. Moreover, the downstream WEE1 target, CDK1, belongs to the 15-gene signature discriminating responders from non-responders across several Asian HCC cohorts treated with TACE.

In a therapeutic perspective, locoregional delivery of miR-22 during TACE may represent a rational approach to simultaneously counteract HIF-1α-mediated metabolic adaptation and promote mitotic catastrophe in highly replicative cancer cells by targeting WEE1. The locoregional delivery of miR-22 may help reduce the risk of adverse events linked to systemic miRNA delivery reported in the first clinical trial in patients with metastatic solid tumors [28]. Moreover, it may prevent possible adverse events, especially in highly proliferating normal tissues [29]. Indeed, WEE1’s dual activity in maintaining genomic stability in normal cells and supporting tumor survival under stress conditions makes its therapeutic targeting complex. Thus, a tumor-selective targeting along with predictive biomarkers may potentially optimize patient treatment. The increase in miR-22 levels immediately after TACE in non-responder patients could represent a possible indication of WEE1 inhibitors associated with TACE retreatment. Adavosertib was shown to have antitumor activity and improve survival in patients with CCNE1-amplified, advanced refractory solid tumors, especially in epithelial ovarian cancer [30]. Indeed, similarly to miR-22 downregulation in HCC cells, overexpression of cyclin E promotes genomic instability by causing DNA replication stress, and WEE1 is essential to prevent massive DNA damage and premature mitotic entry.

In conclusion, our findings suggest miR-22 serum levels variation shortly after TACE as a non-invasive predictive test to complement the clinical practice to be further explored as an easy and cheap biomarker available as soon as 48 h after the procedure. In addition, our preclinical investigations support miR-22 replacement in the context of TACE, as a novel treatment association aimed at increasing treatment efficacy.

## 5. Conclusions

MiR-22 serum levels variation as early as 48 h after TACE appears to be an easy, cheap, non-invasive, promising test to be further validated in HCC patients. In vitro and in vivo models allowed us to dissect molecular events downstream of miR-22 reduced expression in cancer cells, highlighting a WEE1-mediated modulation of proliferation, cell-cycle progression, apoptotic cell death, and DNA repair, all involved in response to TACE-mimicking conditions. Preclinical models also showed that doxorubicin treatment under chemical hypoxia triggers miR-22 secretion, contributing to the HCC resistant phenotype. These findings suggest that miR-22 testing may represent an early predictor of non-response and, in principle, support the selective delivery of miR-22 to HCC nodules during TACE as a novel therapeutic strategy to be considered in patients who do not respond to the first TACE treatment.

## Figures and Tables

**Table 1:** Baseline demographics and clinical characteristics of patients.

Patient’s Characteristics		Bologna 1 Discovery Cohort (*n* = 15)	Bologna 2 Validation Cohort (*n* = 52)	Faenza&IRST Validation Cohort (*n* = 84)
Age (years old)	<65 years old ≥65 years old	6 (40%) 9 (60%)	18 (34.6%) 34 (65.4%)	55 (65.5%) 29 (34.5%)
Gender	M F	10 (66.7%) 5 (33.3%)	36 (69.2%) 16 (30.8%)	62 (73.8%) 22 (26.2%)
ECOG PS	0 1	10 (66.7%) 5 (33.3%)	39 (75%) 13 (25%)	72 (85.7%) 12 (14.3%)
Child-Pugh class	A B C	15 (100%) 0 0	48 (92.2%) 4 (7.7%) 0	75 (89.3%) 9 (10.7%) 0
BCLC stage	0 A B C	4 (26.7%) 11 (73.3%) 0 0	2 (3.8%) 37 (71.2%) 11 (21.2%) 2 (3.8%)	3 (3.6%) 54 (64.3%) 21 (25%) 6 (7.1%)
Etiology CLD	HBV HCV MAFLD Alcohol-related Associated etiologies Unknown	2 (13.3%) 7 (46.7%) 4 (26.6%) 2 (13.3%) 0 0	6 (11.5%) 24 (46.1%) 19 (36.5%) 8 (15.4%) 9 (17.3%) 2 (3.8%)	11 (13.1%) 36 (42.9%) 31 (36.9%) 26 (30.9%) 23 (27.4%) 9 (10.7%)
Nodularity	Uninodular Multinodular	15 (100%) 0	32 (61.5%) 20 (38.5%)	52 (61.9%) 32 (38.1%)
Size (main lesion in multinodular)	≤2 cm 2–3 cm 3–4 cm 4–5 cm >5 cm	6 (40%) 6 (40%) 3 (20%) 0 0	18 (34.6%) 12 (23.1%) 5 (9.6%) 6 (11.5%) 8 (15.4%)	42 (50%) 22 (26.2%) 7 (8.3%) 9 (10.7%) 4 (4.8%)
Portal vein invasion	present	0	0	3 (3.6%)
Extrahepatic spread	present	0	0	0
AFP (ng/mL)	≤20 21–200 ≥201	11 (73.3%) 4 (26.6%) 0	24 (46.1%) 15 (28.8%) 9 (17.3%) NA in 4 cases (7.7%)	54 (64.3%) 9 (10.7%) 5 (5.9%) NA in 16 cases (19%)
Response to treatment	response (3 months) non-response (3 months)	6 (40%) 9 (60%)	29 (55.8%) 23 (44.2%)	46 (54.8%) 38 (45.2%)

M: Male. F: Female. ECOG PS: Eastern Cooperative Oncology Group Performance Status (0–5). BCLC: Barcelona Clinic Liver Cancer staging system. Etiology CLD: Etiology of the underlying Chronic Liver Disease (CLD). HBV: Hepatitis B virus. HCV: Hepatitis C virus. MAFLD: Metabolic- Associated Fatty Liver Disease. AFP: Alfa-feto-Protein. NA: Data not available in clinical records.

## Data Availability

The microarray data generated and analyzed during the current study are available in the NCBI Gene Expression Omnibus (GEO) data repository with the accession number GSE327216.

## Supplementary Materials

The following supporting information can be downloaded at: https://www.mdpi.com/article/10.3390/cells15080722/s1, Figure S1: Validation cohorts and preclinical models; Figure S2: WEE1 target; Table S1: Real-Time PCR and WB.

## Abbreviations

The following abbreviations are used in this manuscript:

HCC	Hepatocellular carcinoma
TACE	Transarterial chemoembolization
HIF1α	Hypoxia-Inducible Factor 1-Alpha
EPO	Erythropoietin
DEB	Drug-eluting bead
PFS	Progression-free survival
OS	Overall survival
HBV	Hepatitis B Virus
GLUT1	Glucose transporter 1
WEE1	WEE1 G2 Checkpoint kinase
CDK1	Cyclin-dependent Kinase 1
PARP	Poly (ADP-ribose) polymerase
EASL	European Association for the Study of the Liver
CT	Computed Tomography
MRI	Magnetic Resonance Imaging
MRECIST	Modified Response Evaluation Criteria in Solid Tumors
BCLC	Barcelona Clinic Liver Cancer
FC	Fold Change
ddPCR	Droplet Digital Polymerase Chain Reaction
TCGA-LIHC	The Cancer Genome Atlas Liver Hepatocellular Carcinoma
ECOG PS	Eastern Cooperative Oncology Group Performance Status
CLD	Chronic Liver Disease
HCV	Hepatitis C Virus
MAFLD	Metabolic Associated Fatty Liver Disease
AFP	Alfa-feto-Protein
PBS	Phosphate Buffer Solution
RT	Room Temperature
BrdU	5-bromo-2′-deoxyuridine
NOD/SCID	Non-Obese Diabetic/Severe Combined Immunodeficiency
ShRNA	Short Hairpin RNA
DMOG	Dimethyloxalylglycine
CoCl_2_	Cobalt (II) Chloride
DMSO	Dimethyl sulfoxide
phospho-γ-H2AX	Phosphorylated histone H2AX
DAPI	4′,6-diamidino-2-phenylindole
ROC	Receiver Operating Characteristics
AUC	Area Under the Curve
R	Responders
NR	Non-responders
CI	Confidence Interval
WT	Wild Type
MUT	Mutated
ATM	Ataxia-telangiectasia mutated
BRCA1	Breast Cancer type 1
OLT	Orthotopic Liver Transplant
DLBCL	Diffuse large B-cells lymphoma
R-CHOP	Rituximab, Cyclophosphamide, Hydroxydaunorubicin (doxorubicin), Oncovin (vincristine), and Prednisone
DEN	Diethylnitrosamine
