# Supplementary material for: Circulating miR-22 Early Predicts TACE Non-Response and Targets WEE1 in Hepatocellular Carcinoma
Source: Cells. 2026 Apr 19;15(8):722. doi: 10.3390/cells15080722 (PMC13114291; doi:10.3390/cells15080722)
Supplement: Supplementary file 1 [file cells-15-00722-s001.zip › S1_Supplementary Tables.pdf]

Supplementary Table 1 – Primer sequences for Real Time PCR

Gene	Primer sequence
**WEE1**	Fw 5'-AAGTTGAAGAGGGCGATAGTC-3'<br>Rv 5'-GCCATTGATCTCCATTTCTCGG-3'
**ß-ACTIN**	Fw 5'-ACCTTCTACAATGAGCTGCG-3'<br>Rv 5'-CCTGGATAGCAACGTACATGG-3'
**WEE1_MIR-22_MUT**	Fw 5'- TTGATGAAA**TAGT**CTATTTGCCTTTTTTTTTTTTTCC-3'<br>Rv 5'-AAGCAGCTATACATTTCAC-3'
**WEE1_sequencing**	Fw 5'-CATGAACCATGGGATGATGA-3'<br>Rv 5'-AAGAGTCCGGGAAGGACATT-3'

Supplementary Table 2 - Antibodies for WB analysis

Antibody	Catalogue number	Company
**WEE1**	#13084	Cell Signaling Technologies
**Beta-actin**	#sc-47778	Santa Cruz
**Phospho-CDK1 (Tyr15)**	#PA1-4617	Thermo Fisher Scientific
**Cleaved caspase-3 (Asp175)**	#9661	Cell Signaling Technologies
**BAX**	#2772	Cell Signaling Technologies
**GAPDH**	#2118	Cell Signaling Technologies
