# Supplementary material for: Circulating miR-22 Early Predicts TACE Non-Response and Targets WEE1 in Hepatocellular Carcinoma
Source: Cells. 2026 Apr 19;15(8):722. doi: 10.3390/cells15080722 (PMC13114291; doi:10.3390/cells15080722)
Supplement: Supplementary file 1 [file cells-15-00722-s001.zip › S2_Supplementary Material.pdf]

TCGA Dataset Analysis

Bioinformatic analyses were done in R software v. 4.2.1. (R Core Team, 2020). Data from The Cancer Genome Atlas Liver Hepatocellular Carcinoma project (TCGA-LIHC) cohort comprising 374 HCC and 50 non-tumor liver samples were retrieved through FirebrowseR (Deng et al., 2017). Tumoral samples were divided based on miR-22 expression levels into high and low expressing groups. Differential expression analysis was performed using the DeSeq2 package (Love et al., 2014) with the low expressing group set as a reference. The Benjamini-Hochberg method was used to adjust the p-value for multiple comparisons. Gene set enrichment analysis (GSEA) was done to further explore the roles of the resulting deregulated genes using the fgsea R package (Korotkevich et al., 2021), run with a list of gene sets compiled from the Human Molecular Signatures Databases (MSigDB) Collections: hallmark gene sets, Gene Ontology, and KEGG (Subramanian et al., 2005; Mootha et al., 2003). Genes of interest were plotted using boxplots. Survival outcomes were obtained from TCGA-CDR (Liu et al., 2019).

References

- R Core Team (2020). — European Environment Agency Available from: https://www.eea.europa.eu/data-and-maps/indicators/oxygen-consuming-substances-in-rivers/r-development-core-team-2006. Accessed August 31, 2022

- Deng M, Brägelmann J, Kryukov I, Saraiva-Agostinho N, Perner S. FirebrowseR: an R client to the Broad Institute's Firehose Pipeline, Database, Volume 2017, 2017, baw160, https://doi.org/10.1093/database/baw160

- Love MI, Huber W, Anders S. Moderated estimation of fold change and dispersion for RNA-seq data with DESeq2. Genome Biol 2014;15:550

- Korotkevich G, Sukhov V, Budin N, Shpak B, Artyomov MN, Sergushichev A. Fast gene set enrichment analysis. 2021;060012

- Subramanian A, Tamayo P, Mootha VK, Mukherjee S, Ebert BL, Gillette MA, Paulovich A, Pomeroy SL, Golub TR, Lander ES, Mesirov JP. Gene set enrichment analysis: A knowledge-based approach for interpreting genome-wide expression profiles. Proc Natl Acad Sci 2005;102:15545–15550

- Mootha VK, Lindgren CM, Eriksson K-F, Subramanian A, Sihag S, Lehar J, Puigserver P, Carlsson E, Ridderstråle M, Laurila E, Houstis N, Daly MJ, Patterson N, Mesirov JP, Golub TR, Tamayo P, Spiegelman B, Lander ES, Hirschhorn JN, Altshuler D, Groop LC. PGC-1alpha-responsive genes involved in oxidative phosphorylation are coordinately downregulated in human diabetes. Nat Genet 2003;34:267–273

- Liu J, Lichtenberg T, Hoadley KA, Poisson LM, Lazar AJ, Cherniack AD, Kovatich AJ, Benz CC, Levine DA, Lee AV, Omberg L, Wolf DM, Shriver CD, Thorsson V; Cancer Genome Atlas Research Network; Hu H. An Integrated TCGA Pan-Cancer Clinical Data Resource to Drive High-Quality Survival Outcome Analytics. Cell. 2018 Apr 5;173(2):400-416.e11. doi: 10.1016/j.cell.2018.02.052. PMID: 29625055; PMCID: PMC6066282.
