# Supplementary material for: Circulating miR-22 Early Predicts TACE Non-Response and Targets WEE1 in Hepatocellular Carcinoma
Source: Cells. 2026 Apr 19;15(8):722. doi: 10.3390/cells15080722 (PMC13114291; doi:10.3390/cells15080722)
Supplement: Supplementary file 1 [file cells-15-00722-s001.zip › S3_Supplementary Figure legends REV_UNTRACKED.pdf]

Supplementary Figure Legends

Figure S1. Evaluation of circulating miR-22 levels in HCC patients undergoing multiple TACE cycles. (**A**) Circulating miR-22 levels in serum samples from two HCC patients collected at different time points before and after TACE (left). Circulating miR-22 levels analyzed in different biological samples from two HCC patients in different Real-Time PCR runs (right). HCC patients are from validation cohort 1. Y-axes report $2^{-\Delta\Delta Ct}$ values corresponding to serum miR-22 levels. Mean values are displayed. Real-Time PCR was performed in triplicate. (**B**) Box plot graph of tumor size (mm) in HCC patients (validation cohort 2) undergoing TACE. Patients are divided in responders (R) and non-responders (NR). Y-axis reports tumor size measurement, the dimensions of the major diameter were reported (mm). Mean ± SD values are displayed. The students' t-test was used for comparison between two groups. (**C**) Contingency graph of serum α-fetoprotein (AFP) levels in HCC patients (validation cohort 2) undergoing TACE. Patients are divided in responders (R) and non-responders (NR). AFP levels (ng/mL) were dichotomized using a cut-off of 20 ng/mL Y-axis reports the percentage of patients with different AFP levels in each group. The Pearson chi-square test was used to evaluate the association between categorical variables. (**D**) Pre-treatment serum miR-22 levels in HCC patients (validation cohort 2) undergoing consecutive TACE cycles. Y-axis reports $2^{-\Delta\Delta Ct}$ values corresponding to circulating miR-22 levels. Mean ± SD values are displayed. The Kruskal-Wallis test was used for comparison of circulating miR-22 levels among the three groups. (**E**) Box plot graph of post-treatment (48 h) serum miR-22 levels in HCC patients (validation cohort 2) undergoing the second TACE cycle (N=23). (**F**) Box plot graph of serum miR-22 fold-change (FC) in HCC patients (validation cohort 2) undergoing the second TACE cycle (N=23). Patients are divided in responders (R, N = 14) and non-responders (NR, N = 9) with respect to the second TACE cycle. Y-axes report $2^{-\Delta\Delta Ct}$ values corresponding to circulating miR-22 levels. (**G**) Correlation plot between basal intracellular miR-22 levels and cell viability in doxorubicin-treated cells (0.5 µM) under hypoxic conditions. Y-axis shows cell viability expressed as the ratio between cell viability in treated and untreated cells (%). X-axis shows log2-transformed $2^{-\Delta\Delta Ct}$ values corresponding to intracellular miR-

22 levels. The Pearson correlation test was used. (**H**) Correlation plot between intracellular miR-22 levels and tumor volume in doxorubicin and DMOG treated xenograft mice. Axes show log2-transformed $2^{-\Delta\Delta Ct}$ values corresponding to intracellular miR-22 levels and log2-transformed tumor volume (mm^3). The Spearman correlation test was used. (**I**) Real-Time PCR analysis of HIF-1α pathway in xenograft mice treated with DMOG (N = 2) or control group (N = 1). Y-axis reports $2^{-\Delta\Delta Ct}$ values corresponding to gene expression levels. Mean ± SD values are displayed. (**J**) Measurement of tumor volume in xenograft mice treated with DMOG (N = 2), DMOG+DOXO (N = 8) or control group (N = 1). Y-axis reports mean tumor volumes (mm^3) in each group. Mean ± SD values are displayed. (**K**) Correlation plot between extracellular and exosomal miR-22 levels in HCC cell lines. Axes show log2-transformed $2^{-\Delta\Delta Ct}$ values corresponding to extracellular and exosomal miR-22 levels. Cel-miR-39 was used as spike-in miRNA. The Pearson correlation test was used.

<u>Figure S2</u>. WEE1 is a target of miR-22 in HCC. (**A**) TargetScan prediction of miR-22-3p hypothetical binding site in WEE1 3’UTR. Stars represent mutated bases in WEE1-3’UTR mutated (MUT) vector. (**B**) Correlation graph between miR-22 and WEE1 mRNA levels in HCC cell lines. Axes report $2^{-\Delta\Delta Ct}$ values corresponding to log2-transformed miR-22 and WEE1 levels. (**C**) Growth curves of control (MZIP-shRNA) and miR-22-silenced (MZIP-22) HepG2 cells treated with WEE1 inhibitor adavosertib (400 nM). Vehicle: DMSO. Growth curves were normalized to T0. Mean ± SD values are reported. (**D**) Volcano plots resulted from differential expression analysis (DEA) analyses comparing HCC patients with high versus low WEE1 expression. Differentially expressed genes (DEGs) are shown in blue; not significantly dysregulated genes are portrayed in grey; and log2 fold change axes are set on −1.5 and +1.5, respectively.
