# Supplementary figures and images for: Circulating miR-22 Early Predicts TACE Non-Response and Targets WEE1 in Hepatocellular Carcinoma
Source: Cells. 2026 Apr 19;15(8):722. doi: 10.3390/cells15080722 (PMC13114291; doi:10.3390/cells15080722)

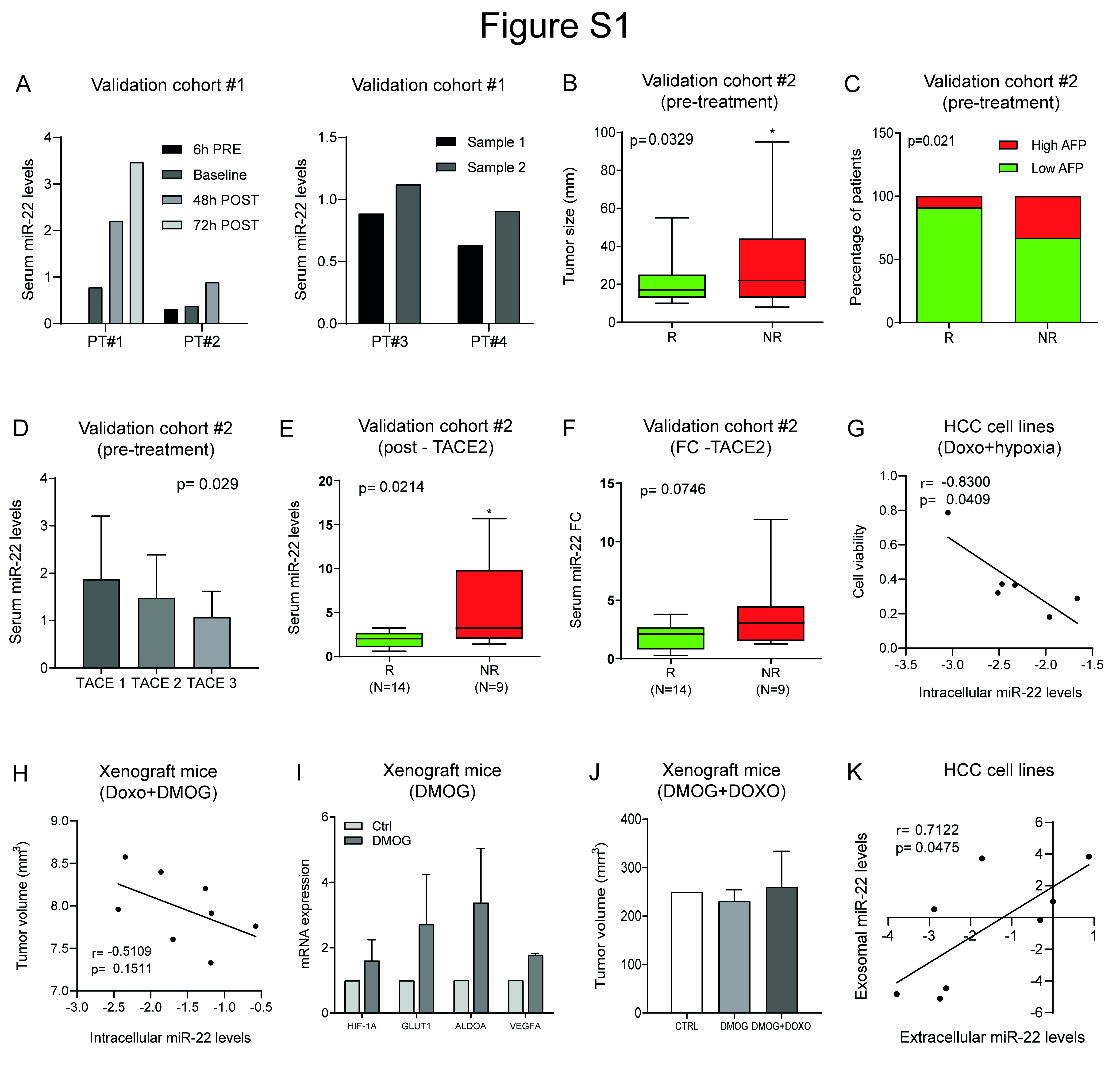

Supplement: Supplementary file 1 [file cells-15-00722-s001.zip › Figure S1 - Validation cohorts -REV.tif]

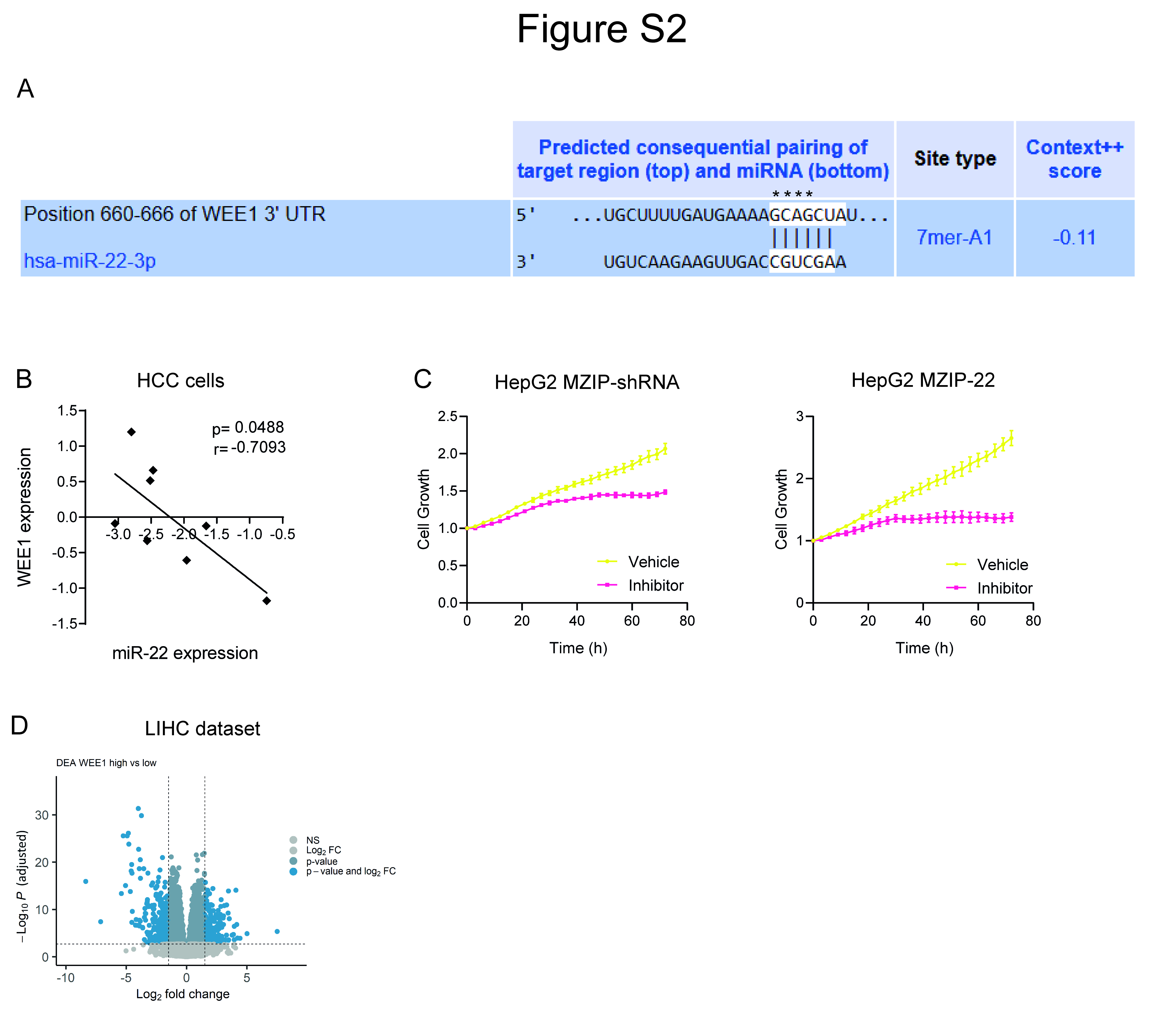

Supplement: Supplementary file 1 [file cells-15-00722-s001.zip › Figure S2 - target WEE1.tif]
